# Variability of definition of high‐risk multiple myeloma across phase III clinical trials

**DOI:** 10.1002/jha2.675

**Published:** 2023-03-28

**Authors:** Faris Jamal Abu Za'nouneh, Obada Ababneh, Carolina Schinke, Sharmilan Thanendrarajan, Maurizio Zangari, John D. Shaughnessy, Fenghuang Zhan, Frits van Rhee, Samer Al Hadidi

**Affiliations:** ^1^ Faculty of Medicine Jordan University Of Science and Technology Irbid Jordan; ^2^ Myeloma Institute University of Arkansas for Medical Sciences Little Rock Arkansas USA

**Keywords:** clinical trials, high risk, multiple myeloma, phase III

## Abstract

The definition of high‐risk multiple myeloma (HRMM) is evolving. Use of a clear definition of HRMM in clinical trials was not previously studied. We explored the definition of HRMM in completed phase III clinical trials. There is extreme variability in the definition and cutoffs used to define HRMM, with a significant number of studies lacking a clear definition. Our study provides a quantification of the variability in defining HRMM and suggests a need to better define HRMM in future clinical trials to enable more consistent treatment recommendations.

## 1 INTRODUCTION

Multiple myeloma (MM) is a plasma cell neoplasm where long‐term disease control is possible [1]. High‐risk multiple myeloma (HRMM) is characterized by poor prognosis with early disease relapse and/or development of refractory disease [2, 3]. Multiple tools were developed for risk stratification which include the revised International Staging System (R‐ISS) that was introduced in 2015, which incorporates serum β‐2 microglobulin, albumin, lactate dehydrogenase, and high‐risk chromosomal abnormalities [del(17p), *t*(4;14), and *t*(14;16)] [4]. It is clear that a number of parameters beyond those included in the R‐ISS also confer a poor prognosis. These include *t*(14;20), non‐hyperdiploidy, chromosome 1q abnormalities, gene expression profiling (GEP), and deletion/inactivation of both copies of p53 [5, 6, 7]. Plasma cell leukemia, extramedullary disease, circulating plasma cells (>0.02%), and the presence of at least three large focal lesions (>5 cm^2^) are associated with poor outcomes [8, 9, 10]. Patient‐related factors including the presence of renal failure and frailty are also associated with worse outcomes [11]. A clear definition of HRMM has not been widely accepted nor uniformly applied, possibly due to differences in data in the literature suggesting that certain chromosomal abnormalities and other factors may confer different levels of risk. In this study, we investigated the variability of definitions of HRMM and frequencies of cytogenetics reporting used across interventional phase III MM clinical trials. Identification of variability of HRMM definition is an important step toward a standardized definition that can ultimately help in better understanding of patients’ outcomes and in designing specific clinical trials focused on HRMM.

## 2 METHODS

The ClinicalTrials.gov database was searched to identify all registered phase III interventional MM trials. We searched the ClinicalTrials.gov database for the terms “*myeloma*,” “*multiple myeloma*,” and “*plasma cell dyscrasia*” and included all interventional phase III trials. Smoldering myeloma trials and trials that investigated supportive care in MM were excluded. The study protocol, data available on ClinicalTrials.gov, and all subsequent publications related to included clinical trials were used for data extraction. Data, including frequencies of cytogenetics reporting, definitions of HRMM, staging criteria, number of enrolled patients, trial start date and publication date, source of funding, and location of the study, were collected. Data cutoff was June 2022. Trials initial start date ranged from 1993 to 2022. Data collection was done in July 2022. To identify the factors associated with the HRMM definition, the Mann–Whitney *U* test was used to compare continuous variables, whereas the chi‐square test was used to compare categorical variables. The Spearman correlation test was used to study contentious factors associated with missing cytogenetic percentages. Binary logistic regression was used to identify factors associated with the HRMM definition. Given the publicly available data, the study was exempt from the University of Arkansas for Medical Sciences institutional review board review.

## 3 RESULTS

The search retrieved a total of 327 trials of which 56 studies were excluded due to interventions targeting MM symptoms and/or treatment of adverse events such as infection management. Our final analysis included 271 trials (Supporting Information). Only 96 trials defined HRMM (35%), with the cytogenetics‐based definition being the most common (96%, *n* = 92/96). Other trials also used ISS, whereas five trials used GEP. Of the 28 trials that have commenced as the R‐ISS was defined in 2015 [3], 4 trials used R‐ISS criteria. Ten trials started before 2015 that retrospectively reported the use of R‐ISS staging system in their peer‐reviewed publications. A total of 79 trials were conducted after 2005, in which R‐ISS was used in 14 trials (17.7%) and ISS was used in 50 trials (63.3%). A total of 54 trials(56.3%) included only patients with newly diagnosed MM. Table 1 shows the characteristics of the included trials. As shown in Table 2, del(17p), *t*(4;16), and *t*(14;16) were the most reported aberrations although the reporting of thresholds used to define positivity was only reported in 18%, 17.6%, and 18.2, respectively. Missing cytogenetics data were reported in 66 trials with a median of 22.6% of patients per trial having missing cytogenetics data (IQR: 36.7%). A trend of lower missing cytogenetics in more recent trials was noted. Peer‐reviewed publications, a larger patient enrollment, and more recent enrollment and publication dates were all significantly associated with HRMM definition reporting (*p* < 0.001). However, the location of the study (United States vs. others) and funding source (industry vs. nonindustry) did not predict whether the definition of HRMM was reported (*p* = 0.071 and 0.452, respectively).

**TABLE 1:** Characteristics of the trials defining high‐risk multiple myeloma (HRMM) based on the trial start date

	Total	Before 2005	Between 2005 and 2014	After 2014
Number of trials that defined HRMM	96/271	13/65	55/120	28/86
Criteria that were used in the definition				
Cytogenetics	82 (85.4%)	8 (61.5%)	48 (87.3%)	26 (92.9%)
Cytogenetics, B2M	3 (3.1%)	2 (15.4%)	0 (0%)	1 (3.6%)
Cytogenetics, GEP	3 (3.1%)	1 (7.7%)	2 (3.6%)	0 (0%)
Cytogenetics, ISS	3 (3.1%)	1 (7.7%)	2 (3.6%)	0 (0%)
Cytogenetics, ISS, LDH	1 (1%)	1 (7.7%)	0 (0%)	0 (0%)
ISS	2 (2.1%)	0 (0%)	1 (1.8%)	1 (3.6%)
GEP	2 (2.1%)	0 (0%)	2 (3.6%)	0 (0%)
Treatment status				
Newly diagnosed	54	10	33	11
R/R	42	3	22	17
Peer‐reviewed publication				
Yes	85	12	52	21
No^a^ (data from protocol)	11	1	3	7
Median enrollment patient number per trial (IQR)	543 (545)	450 (260)	493 (459)	346 (246)
Median start date (IQR)	2011 (7)	2002 (3)	2010 (5)	2017 (2.25)
Median publication year (IQR)	2017 (7)	2009 (3)	2017 (4.25)	2020 (2)
Source of funding				
Industry	63	1	38	24
Nonindustry	33	12	17	4
Location of the study				
International trials including the US	28	0	16	12
Single‐center trials in the US	6	2	4	0
Multi‐centric trials in the US	13	3	8	2
Trials outside the US	49	8	27	14
Staging criteria^b^				
Durie–Salmon	6	3	3	0
ISS	61	8	38	15
R‐ISS	13	0	10	4
Not reported	25	2	12	11
Mean % of missing cytogenetics (SD)	27.1% (22.1)	51.5% (26.1)	24.9% (19.5)	19.2% (17.2)

Abbreviations: GEP, gene expression profile; IQR, Interquartile range; ISS, International Staging System; LDH, lactate dehydrogenase; R‐ISS, revised International Staging System; R/R, relapsed/refractory; SD, standard deviation.

^a^ Data were obtained from either the protocol or ClinicalTrials.gov.

^b^ 11 trials used more than one staging criteria.

**TABLE 2:** Reported cytogenetics aberrations

Cytogenetic abnormality	*t*(4;14)	*t*(14;16)	del(17p)	*t*(14;20)	amp 1q	del13q	del(1p32)
Studies (%) (*n* = 92)	85 (92.4%)	66 (71.7%)	89 (96.7%)	8 (8.6%)	15 (16.3%)	12 (13%)	5 (5.4%)
Studies defining cutoff	15	12	16	0	2	0	0
Median cutoff of the % of cytogenetic abnormality (IQR)	15 (26)	10 (27)	20 (45)	n/a	n/a	n/a	n/a

Abbreviations: IQR; Interquartile range, n/a; not available/applicable.

None of these factors were significant in the binary logistic regression model (*p* > 0.05). Finally, both a later starting date of the trial and the publication year were correlated significantly with a lower missing cytogenetics data (*p* < 0.05).

## 4 DISCUSSION

The findings of our study suggest that the definition of HRMM is underreported in interventional phase III MM trials. Although almost all the trials that defined HRMM used a cytogenetics‐based definition, a specific threshold and/or exact definitions were lacking. Such information is important as different high‐risk cytogenetics aberrations can influence the disease progression and response to treatment differently [12]. For example, patients with *t*(4;14) and del(17p) may benefit from prolonged proteasome inhibitor treatment [13, 14].

The definition of HRMM has evolved over time to integrate both clinical and cytogenetic features. We found that 13.5% of trials used the recent R‐ISS staging criteria. Although 14.3% of trials started in recent years used R‐ISS as staging criteria, 10 trials (18.2%) published between 2005 and 2014 have reported the R‐ISS stage of their patients in their subsequent publications. This suggests that R‐ISS is becoming more appealing to different primary investigators compared to the ISS and Durie–Salmon staging criteria. However, the lack of consistent definitions and variations in HRMM definitions pose a significant challenge in making accurate clinical decisions and drawing valid conclusions from clinical trials [15].

The prognosis for the HRMM remains poor [16]. The goal is to develop novel treatment approaches targeting HRMM; however, the lack of a unified definition of which patients are classified as HRMM makes it difficult to design and/or directly compare survival outcomes between clinical trials focused on HRMM. Our study found a significant amount of missing cytogenetics data in phase III clinical trials. The prognostic value of missing and/or unsuccessful cytogenetics in MM is unknown and almost all the included trials in this study did not mention the reason for missing cytogenetics. Using other techniques like GEP, comparative genomic hybridization, single nucleotide polymorphism arrays, and next‐generation sequencing can be considered for patients with unknown cytogenetics [17].

We also observed the use of different thresholds in determining the presence of cytogenetic abnormality. The presence of del(17p) is not prognostic in all patients, and the size of the clone carrying the abnormality is important [18, 19]. Patients presenting with del(17p) less than <60% of their plasma cells did not show a specific poor outcome. What is even more problematic is the underreporting of the used threshold in phase III clinical trials. High inter‐lab and intra‐lab variabilities in cytogenetics testing by different labs were previously reported [20]. The number of cytogenetic abnormalities is important. In MASTER trial [21], the presence of two or more cytogenetic abnormalities (defined as ultrahigh‐risk disease) was associated with worse outcomes, which did not occur in patients with zero or one cytogenetic abnormalities. The GMMG‐CONCEPT trial investigated isatuximab plus KRd in HRMM patients based on the presence of at least one cytogenetic abnormality [22]. The OPTIMUM/MUKnine trial also used the same high‐risk stratification criteria as the MASTER trial but only included ultrahigh‐risk patients and primary plasma cell leukemia patients [23]. Although cytogenetics‐based HRMM definition is the most common criteria to be used in HRMM‐only trials, GEP is another robust non‐cytogenetics‐based criterion that is commonly used in Total Therapy trials by the University of Arkansas for Medical Sciences group. TT5 only included patients with high‐risk GEP profiles [24]. In these patients with high‐risk GEP profiles, cytogenetic abnormity was not associated with survival disadvantage.

It is important to note that the definition of HRMM continues to evolve. A second revision (R2‐ISS) classified patients into four risk groups that predict different survival outcomes. Compared with the R‐ISS, the new R2‐ISS includes the independent poor prognostic factors 1q gain (3 copies of 1q) or amplification (≥4 copies of 1q) [25]. It is expected that the definition of HRMM will continue to evolve. To improve reporting of the outcomes of phase III clinical trials in HRMM, it is crucial to have a standardized definition for HRMM. This definition should be straightforward to implement, readily accessible, and considered a standard procedure for all patients newly diagnosed with MM.

In conclusion, the majority of studies lacked a clear definition of HRMM and there is extreme variability in the definition of HRMM in phase III clinical trials. In addition, nearly one‐quarter of the patients had an unknown cytogenetics profile in clinical trials which raises concerns of a likely higher proportion of missing data in real‐world. The need for a unified definition, constant reporting, and ongoing innovative clinical trial designs attempting to define HRMM are crucial as this subset of patients requires individualized treatment options with the aim of improving upon their currently poor outcomes.

## AUTHOR CONTRIBUTIONS

Samer Al Hadidi conceived the research idea. Faris Jamal Abu Za'nouneh, Obada Ababneh, and Samer Al Hadidi performed the initial literature review, collected data, performed the statistical analysis, and wrote the initial draft of the manuscript. Carolina Schinke, Sharmilan Thanendrarajan, Maurizio Zangari, John D. Shaughnessy, Fenghuang Zhan, and Frits van Rhee provided critical input on the methodology and analysis. All authors reviewed and approved the final version of the manuscript.

## CONFLICT OF INTEREST STATEMENT

None of the authors has a relevant conflict of interests.

## PREVIOUS PRESENTATION

Portions of this work were presented at the 2022 annual meeting of the American Society of Hematology on December 10–13th in New Orleans, LA.

## CLINICAL TRIAL REGISTRATION

The authors have confirmed clinical trial registration is not needed for this submission.

## PATIENT CONSENT STATEMENT

The authors have confirmed patient consent statement is not needed for this submission.

## ETHICS STATEMENT

The authors have confirmed ethical approval statement is not needed for this submission.

## Supporting information

Supporting Information

## Data Availability

The data that support the findings of this study are available from the corresponding author upon reasonable request.
