# Supplementary material for: Variability of definition of high‐risk multiple myeloma across phase III clinical trials
Source: EJHaem. 2023 Mar 28;4(2):454–8. doi: 10.1002/jha2.675 (PMC10188470; doi:10.1002/jha2.675)
Supplement: Supplementary file 1 — Supporting Information [file JHA2-4-454-s001.pdf]

Number	NCT Number
1	NCT04975997
2	NCT01568866
3	NCT03539744
4	NCT02541383
5	NCT03651128
6	NCT01971658
7	NCT01734928
8	NCT00560053
9	NCT01208766
10	NCT05257083
11	NCT02248428
12	NCT02576977
13	NCT04923893
14	NCT04162210
15	NCT05317416
16	NCT03110562
17	NCT00038233
18	NCT04484623
19	NCT05020236
20	NCT00452569
21	NCT01080391
22	NCT01801436
23	NCT03993912
24	NCT00622336
25	NCT02362165
26	NCT01002248
27	NCT02659293
28	NCT04246047
29	NCT02215980
30	NCT04288765
31	NCT01712789
32	NCT02136134
33	NCT00891384
34	NCT00048230
35	NCT02076009
36	NCT05243797
37	NCT00057564
38	NCT05028348
39	NCT01296503
40	NCT02252172
41	NCT01070862
42	NCT04483739
43	NCT05083169
44	NCT01532856
45	NCT04287660
46	NCT01850524
47	NCT01908621

48	NCT00075829
49	NCT00218855
50	NCT03748953
51	NCT03729804
52	NCT02195479
53	NCT03277105
54	NCT03710603
55	NCT04649060
56	NCT04181827
57	NCT02516696
58	NCT01470131
59	NCT00111319
60	NCT02755597
61	NCT03234972
62	NCT02726581
63	NCT03652064
64	NCT00378222
65	NCT02916420
66	NCT02811978
67	NCT00367185
68	NCT00005834
69	NCT02739594
70	NCT03180736
71	NCT03217812
72	NCT01324947
73	NCT00424047
74	NCT02579863
75	NCT01109004
76	NCT01311687
77	NCT00644306
78	NCT00813150
79	NCT00416273
80	NCT02412878
81	NCT02197221
82	NCT02312258
83	NCT00689936
84	NCT01266811
85	NCT00063726
86	NCT01091831
87	NCT00934154
88	NCT00416208
89	NCT01023308
90	NCT01818752
91	NCT01093196
92	NCT04751877
93	NCT01302392
94	NCT01146834
95	NCT00056160

96	NCT00480363
97	NCT01530594
98	NCT03952091
99	NCT01090089
100	NCT01621672
101	NCT01335399
102	NCT03158688
103	NCT03836014
104	NCT00171925
105	NCT03301220
106	NCT00097981
107	NCT01063179
108	NCT00928486
109	NCT00657553
110	NCT00002548
111	NCT00083564
112	NCT00179647
113	NCT01134484
114	NCT00722566
115	NCT03901963
116	NCT00103506
117	NCT00432458
118	NCT00773747
119	NCT00376883
120	NCT00083876
121	NCT01309334
122	NCT03742297
123	NCT00732641
124	NCT03720041
125	NCT00910897
126	NCT00443235
127	NCT00461747
128	NCT04939142
129	NCT00420849
130	NCT03562169
131	NCT00571168
132	NCT03617731
133	NCT01301963
134	NCT00083551
135	NCT01554852
136	NCT00006232
137	NCT00002878
138	NCT03859427
139	NCT00103662
140	NCT00014339
141	NCT01564537
142	NCT00546780
143	NCT00028886

144	NCT03029234
145	NCT00064038
146	NCT01685814
147	NCT02406144
148	NCT00747877
149	NCT00049673
150	NCT00033332
151	NCT00478777
152	NCT03891914
153	NCT00008229
154	NCT00405756
155	NCT00003603
156	NCT05006469
157	NCT00017602
158	NCT05236621
159	NCT01102426
160	NCT00434161
161	NCT01891643
162	NCT00004165
163	NCT03792620
164	NCT00602511
165	NCT03402295
166	NCT02495922
167	NCT00232934
168	NCT02024815
169	NCT00002678
170	NCT00256776
171	NCT00551928
172	NCT00002653
173	NCT02181413
174	NCT00417911
175	NCT01413178
176	NCT00892346
177	NCT01241708
178	NCT00734877
179	NCT01239797
180	NCT01916252
181	NCT00872352
182	NCT01160380
183	NCT03151811
184	NCT04096066
185	NCT00002599
186	NCT00633542
187	NCT00217438
188	NCT00522392
189	NCT03275285
190	NCT04270409
191	NCT00270101

192	NCT03143049
193	NCT02990338
194	NCT00002850
195	NCT01910987
196	NCT00200681
197	NCT00932217
198	NCT02112175
199	NCT01863550
200	NCT00002556
201	NCT04071457
202	NCT01539083
203	NCT00546988
204	NCT03246529
205	NCT00573391
206	NCT00950768
207	NCT00416897
208	NCT02288741
209	NCT00602641
210	NCT03941860
211	NCT00205764
212	NCT04566328
213	NCT02575144
214	NCT00207805
215	NCT00083915
216	NCT00222053
217	NCT00507416
218	NCT01208662
219	NCT03440411
220	NCT00215943
221	NCT00574080
222	NCT00344422
223	NCT03948035
224	NCT00644228
225	NCT01191060
226	NCT00098475
227	NCT04549363
228	NCT00111748
229	NCT00001561
230	NCT03937635
231	NCT02322320
232	NCT00093028
233	NCT00657488
234	NCT02419118
235	NCT03759093
236	NCT00177047
237	NCT00572169
238	NCT02145598
239	NCT00430365

240	NCT03357952
241	NCT04934475
242	NCT00038090
243	NCT00114101
244	NCT00514371
245	NCT02066454
246	NCT00998270
247	NCT03319667
248	NCT00090493
249	NCT00838357
250	NCT04459416
251	NCT00984828
252	NCT00415987
253	NCT01345019
254	NCT02336386
255	NCT01169337
256	NCT03428373
257	NCT00205751
258	NCT02155634
259	NCT04513639
260	NCT00219258
261	NCT00861250
262	NCT00477971
263	NCT03595800
264	NCT00330759
265	NCT00950911
266	NCT01016548
267	NCT00003341
268	NCT02270307
269	NCT00869206
270	NCT01208818
271	NCT00145652
